# Hepatitis C Virus and Nonliver Solid Cancers: Is There an Association between HCV and Cancers of the Pancreas, Thyroid, Kidney, Oral Cavity, Breast, Lung, and Gastrointestinal Tract?

**DOI:** 10.1155/2017/8349150

**Published:** 2017-05-03

**Authors:** Saad Qadwai, Tayyaba Rehman, Jonathan Barsa, Zeeshan Solangi, Edward Lebovics

**Affiliations:** ^1^New York Medical College and Westchester Medical Center, 100 Woods Road, Valhalla, NY 10595, USA; ^2^Bristol-Myers Squibb, 5 Research Pkwy, Wallingford, CT 06492, USA

## Abstract

Hepatitis C virus (HCV) is known for its oncogenic potential and has been found to be associated with hepatocellular carcinoma (HCC) and non-Hodgkin lymphoma. It has also been postulated that HCV may play a role in the development of other extrahepatic solid tumors of other organs of the body since it has been isolated from the vessel wall, kidney, and oral mucosa. In this article, we have reviewed epidemiological studies that have been done to look into the relationship of HCV with nonliver solid cancers of the pancreas, thyroid, renal, oral cavity, breast, and lung and nonpancreatic gastrointestinal cancers. Based on this review, HCV might be associated with an increased risk of renal cell and lung cancers.

## 1. Introduction

Approximately 180 million people worldwide are infected by hepatitis C virus (HCV) including almost three million people in the US [1, 2]. HCV virus is known for its oncogenic potential and has been found to be associated with hepatocellular carcinoma (HCC) [3] and non-Hodgkin lymphoma [4]. It has also been postulated that HCV may play a role in the development of extrahepatic solid tumors of other organs of the body since it has been isolated from the blood vessel wall, kidney, and oral mucosa [5–7]. The possible oncogenic effects of HCV can be through various molecular, genetic, and environmental mechanisms. These mechanisms include complement-mediated tissue injury, inhibition of lymphocyte-mediated apoptosis, and mutation of somatic genes like proto-oncogenes or tumor suppressor genes [5, 8–21]. In this article, we have reviewed epidemiological studies that have evaluated the relationship of HCV with nonliver solid tumors.

## 2. Method

We conducted a MEDLINE search of published articles to identify epidemiologic studies on the potential association between HCV infection and solid malignancies other than HCC. We focused on the following malignancies: (1) pancreatic cancer (PAC), (2) thyroid cancer (TC), (3) renal cancer (RC), (4) oral cancer (OC), (5) breast cancer (BC), (6) lung cancer (LC), (7) esophageal cancer (EC), (8) stomach cancer (SC), and (9) colorectal cancer (CRC).

The inclusion criteria of our analysis were as follows:

1. Case-control and cohort study designs focusing on adult population
2. Full-text publications and peer-reviewed articles in the English language
3. Studies that described diagnostic testing for the HCV infections in the participants, for example, enzyme-linked immunosorbent assay (ELISA) and polymerase chain reaction (PCR) for the presence of HCV RNA.

The exclusion criteria of our analysis were case reports and case series.

The MEDLINE search of articles on the association of hepatitis C and nonliver cancers produced the following citations: (1) PAC-: 144; (2) TC-: 65; (3) RC-: 239; (4) OC-: 179; (5) BC-: 111; (6) LC-: 185; (7) EC-: 91; (8) SC-: 93; and (9) CRC-: 118. We screened the potentially relevant studies and, in accordance with predefined criteria, we identified and considered in our systematic review the following number of studies on the association of hepatitis C and nonliver cancers: (1) PAC: 3 case-control studies and 7 cohort studies; (2) TC: 5 case-control studies and 7 cohort studies; (3) RC: 3 case-control studies and 4 cohort studies; (4) OC: 2 case-control studies and 4 cohort studies; (5) BC: 2 case-control studies and 5 cohort studies; (6) LC: 2 case-control studies and 4 cohort studies; and (7) esophagus/stomach/colon/rectal: 2 case-control studies and 5 cohort studies.

## 3. Results

### 3.1. Pancreatic Cancers

In Woo et al., anti-HCV seropositivity was found to be associated with increased risk of developing PAC [22]. In contrary, Chang et al. and Hassan et al. did not find HCV infection to be associated with an increased risk of PAC [23, 24] (Table 1).

In a population-based cohort study, Abe et al. did not find HCV to be associated with increased risk of PAC as compared to individuals without a positive infection marker in a 324,394 person-years follow-up [25]. Huang et al. conducted a nationwide cohort study in Sweden using a National Surveillance Database from 1990 to 2006 and followed to the end of 2008 [26]. The PAC risk in the study population was compared with the general population [26]. In a total of 340,819 person-years in the HCV cohort, 34 PACs were identified [26]. It was noted that HCV slightly increases the risk of pancreatic cancer as compared to general population [26]. El-Serag et al., conducted a cohort study including 146,394 HCV-infected and 572,293 HCV-uninfected patients who received care at Veterans Affairs health care facilities between 1996 and 2004 [27]. In the 1.37 million person-years of follow-up, HCV-associated risk of PAC was slightly elevated, which was attenuated after adjusting for alcohol use, pancreatitis, cholelithiasis or choledocholithiasis, HBV, or primary sclerosing cholangitis [27]. An increased risk of PAC was also noticed among HCV-infected patients in Omland et al. [35]. However, Amin et al. did not show an increased incidence of PAC in HCV-diagnosed patients [28] (Table 2).

### 3.2. Thyroid Cancers

Five case-control studies completed in Italy by Antonelli et al. and Montella et al. have shown consistently increased prevalence and risk of TC among patients diagnosed with HCV infection (Table 3) [29–33]. Antonelli et al. noted six cases of TC in 308 HCV (+) patients, while no TC patient was noted in 616 healthy subjects from iodine-deficient areas (*P* 0.001) and only one TC was noted in 616 healthy subjects from iodine-sufficient areas (*P* 0.003) [29]. Montella et al. showed a statistically significant association between HCV and papillary TC [30]. In another case control study, Antonelli et al. compared the prevalence of TC among HCV-related mixed cryoglobulinemic patients to a five sex-matched control group of subjects aged > 50 years and who had undergone thyroid ultrasound. They noticed two cases of papillary TC in patients with HCV-related mixed cryglobinemia (2/94), while no case was observed among controls (0/582) (*P* 0.001) [31]. In another case-control study, Montella et al. found an association between HCV and TC in overall population and subjects ≥ 50 years [32]. In another study, Antonelli et al. found 3 incidences of TC on fine-needle aspiration (FNA) biopsy of HCV (+) cases while no TC was noted in HCV (−) control group (*P* 0.004) [33] (Table 3). In contrary to the case control studies, none of the cohort studies done in the United States, Australia, Denmark, Sweden, and Taiwan showed an association between HCV and TC [4, 28, 34–37] (Table 4).

### 3.3. Renal Cancers

Gonzalez et al. screened for anti-HCV antibodies and HCV RNA in patients with suspected RC and colon cancer (controls) [38]. They found increased detection of anti-HCV and HCV RNA in RC cases as compared to colon cancer controls (Table 5) [38]. Malaguarnera et al. compared the prevalence of HCV infection in cancer patients to volunteers (controls) [39]. Increased prevalence of anti-HCV was found in patients with RC cases as compared to control group [41]. However, Budakoğlu et al. found no significant increase in HCV positivity in RC patients compared to healthy control group [40] (Table 5).

In a cohort study, Gordon et al. HCV tested cases between 1997 and 2006 were followed for the development of RC until April 2008. The adjusted hazard ratio (HR) for age, African-American race, male gender, and chronic kidney disease (CKD) was 1.77 (95% CI 1.1–2.9, *P* 0.03) [41]. In contrary, another nationwide registry-based cohort study conducted in Sweden on individuals with chronic HCV infection diagnosed between 1990 and 2006 with a mean follow-up time of 9.3 years did not show increased standardized incidence rate (SIR) for RC in this population. Also, SIR was not found to be significantly increased in Omland et al. and Amin et al. [28, 35] (Table 6).

### 3.4. Oral Cancers

Nagao et al. demonstrated both significantly increased detection of anti-HCV antibodies and HCV RNA in patients with OC as compared to control group. Also, significantly decreased prevalence of anti-HCV antibodies was noticed in patients with SC as compared to OC patients [43]. Takata et al. found increased prevalence of HCV antibody in patients with OC as compared to patients with impacted teeth but the association reversed after age adjustment [44] (Table 7).

In a prospective study done by Su et al., incidence of OCs was 2.28-fold higher (6.15 versus 2.69 per 10,000 person-years) among patients with HCV than those in the nonviral hepatitis group (HR 1.9, 95% CI 1.2–3.0) [45]. The positive association was highest among individuals in the 40–49 year age group (HR 2.57, 95% CI 1.2–5.4) [45]. However, Amin et al. did not show an increased association of HCV with cancers of the mouth, tongue, and tonsil [28]. However, Swart et al. showed an increased incidence of cancers of the mouth in patients found to be HCV-positive [36] (Table 8).

### 3.5. Breast Cancers

Su et al. conducted a nationwide population-based case-control study in Taiwan [46] in which 1958 patients with newly diagnosed BC were identified from the National Health Insurance Research Database (NHIRD) between 2000 and 2008 [46]. A randomly selected sample of age-matched cohort of 7832 subjects without cancer was selected for comparison [46]. Three percent of BC subjects had HCV and 2.3% non-BC subjects had HCV [46]. No significant association between HCV infection and BC was found (adjusted OR 1.2, 95% CI 0.9–1.7) after adjusting for area, occupation, urbanization, and income [46]; however, age < 50 years was found to be associated with a 2-fold greater risk of developing BC (OR 2.0, 95% CI 1.2–3.3) among HCV-infected persons [46]. A single-center case control study was performed in France by Larrey et al. in which females aged ≥ 20 years with present and/or past history of chronic HCV infection based on detection of serum anti-HCV antibodies, HCV RNA by PCR, and liver biopsy consulting in an outpatient liver unit for 1 year [47] were included. The control group included female patients with other chronic liver diseases: chronic hepatitis B, alcoholic liver disease, autoimmune hepatitis, hemochromatosis, nonalcoholic liver disease, and chronic cholangitis. The results of this study noted a higher prevalence of BC in the HCV group (17/294, 6%, 95% CI 3.1–8.4), but there was no significant statistical difference with the control group (5/107, 5%, 95% CI 0.7–8.7) [47]. Furthermore, no significant difference in BC was found among HCV and control group for females younger than 40 years (0/28 versus 0/12), between 41 and 60 years (5/146, 3.4%, 95% CI 0.5–6.4 versus 1/55, 2%, 95% CI 0–5.3), and older than 60 (12/120, 10%, 95 CI 4.6–15.4 versus 4/40, 10%, 95% CI 0.7–19.3) [47].

HCV was not found to be associated with an increased incidence of BC in cohort studies conducted by Allison et al. (SRR 0.7, 95% CI 0.6–0.8), Omland et al. (SIR 0.25, 95% CI 0.03–0.9), Amin et al. (SIR 0.4, 95% CI 0.3–0.5), and Swart et al. (SIR 0.4, 95% CI 0.1–0.8) [28, 34–36].

### 3.6. Lung Cancers

In a Turkish retrospective study conducted by Uzun et al., anti-HCV antibodies were tested in 45 patients with LC, 80 patients with benign lung disease, and 135 healthy controls [48]. Detection of anti-HCV abs were significantly higher (*P* < 0.05) among LC patients (3/45) as compared to patients with benign lung disease (0/80) and the healthy controls (1/135) [48]. In another case control study conducted by Malaguarnera et al., increased prevalence of anti-HCV was found in patients with LC (8/22 versus 30/300) as compared to those in the control group (36% versus 10%, *P* < 0.05) [39].

In Allison et al., HCV infection was found to be associated with a slightly increased incidence of LC (SRR 1.6, 95% CI 1.3–1.9) [34]. Similarly, in Swart et al., increased incidence of cancer of trachea, bronchus, and lung were noted (SIR 4.6, 95% CI 2.8–7.1) among subjects infected with HCV [36]. However, in Omland et al., HCV was not found to be associated with increased incidence of lung cancer (SIR 1.95, 95% CI 0.93–3.58) [35]. Similarly, in Amin et al., HCV was not found to be associated with the increased incidence of cancer of the larynx (SIR 1.0, 95% CI 0.4–2.3) and trachea (SIR 1.2, 95% CI 0.9–1.5) [28].

### 3.7. Gastrointestinal (GI) Cancers

Increased risk of EC among HCV-infected population was not observed in the studies done by Amin et al. (SIR 0.5, 95% CI 0.2–1.4) [28], Allison et al. (SRR 2.1, 95% CI 0.9–3.2) [34], and Omland et al. (SIR 1.6, 95% CI 0.04–9.2) [35]. Also, no statistically significant association was found between HCV and SC in Amin et al. (SIR 1.4, 95% CI 0.9–2.1) [28] and Allison et al. (SRR 1.1, 95% CI 0.5–1.6) [34]. A case-control study by Malaguarnera et al. found an increased prevalence of anti-HCV antibodies in patients with colorectal cancer as compared to controls (8/22 (36%) versus 30/300 (10%), *P* < 0.05) [39].

In Allison et al., an increased incidence of rectal cancer was also found in patients with chronic HCV infection (SRR 2.1, 95% CI 1.3–2.8) [34], but colon cancer incidence rate did not increase in chronic HCV-infected patients (SRR 0.4, 95% CI 0.3–0.6) [34]. Colon cancer incidence rate was also not increase in HCV-infected patients in studies by Amin et al. (SIR 0.6, 95% CI 0.5–0.9) [28] and Omland et al. (SIR 1.0, 95% CI 0.2–2.9) [35]. Similarly, no increased incidence of rectal cancer was observed in HCV patients in Amin et al. (SIR 0.3, 95% CI 0.2–0.6) [28] and Omland et al. (SIR 1.8, 95% CI 0.4–5.4) [35]. Furthermore, no increased incidence of CRC was observed in patients with HCV notification (SIR 0.9, 95% CI 0.4–1.8) in Swart et al. [36].

## 4. Discussion

HCV has a hepatotropic potential and is well known for causing hepatocellular carcinoma. Since both liver and pancreas share common endodermal origin, it is postulated that HCV may replicate in pancreatic cells as well [49, 50]. Thus, HCV may play a role in the development of PAC. However, most of the epidemiological studies have not shown an association between the HCV and PAC. Among these, two case-control studies and one cohort study were conducted in Asian population [22, 23, 25]. Only one case-control study done in Korea showed an increased risk of PAC in HCV-positive subjects [22]. On the other hand, focused cohort studies which have not utilized administrative data have not shown a positive association between HCV and PAC [25, 27]. Based on current information, increased PAC screening among HCV-infected patients is not clearly indicated.

Antonelli et al. and Montella et al. collaborated to conduct five case-control studies on the association of HCV and TC in Italy. They have consistently shown an increased association between HCV and TC [29–33]. Although they did not adjust for some of the TC risk factors in their analyses such as history of radiation exposure, they were still able to show increased risk of TC among HCV (+) population as compared to controls from iodine-deficient areas [29] and among population ≥ 50 years [32]. However, no cohort study has been done in Italy to confirm this association between HCV and TC. None of the cohort study done in other parts of the world has shown any significant relationship between HCV and TC [4, 28, 34, 35, 37]. It could be possible that a high prevalence of HCV in certain parts of the world may result in an increased risk of developing TC which could be further explained by genetic and environmental factors. Until now, the data does not clearly support an increase in TC screening among HCV-infected patients.

According to a study utilizing bioinformatics network analysis technique, NY-REN-54 may be the protein responsible for a cause-outcome relationship between HCV and RCC as this protein is common to both [51]. This protein causes disturbance of autophagic response due to ubiquitin-protein ligase-related mechanism [51]. Among three case-control studies, two studies, that is, Gonzalez et al. and Malaguarnera et al., have shown an increased risk of RCC in HCV (+) population [38, 39]. These studies confirmed the HCV status of their subjects by testing for HCV RNA [38, 39]. Moreover, Gonzalez et al. used CRC patients as controls in their study who are expected to have a higher risk of acquiring HCV infection due to frequent hospitalizations and medical procedures as compared to the general population [38]. The result of the study still showed statistically increased prevalence of anti-HCV antibodies and HCV RNA in the RCC patients as compared to the CRC patients [38, 39]. Gordon et al. conducted a follow-up cohort study to the findings of Gonzalez et al. and confirmed that HCV increases the risk of RCC [38, 41]. Based on current data, HCV could be associated with increased risk of RCC. Clinicians should consider screening newly diagnosed RC cases for HCV virus. Also, low threshold should be maintained for screening RC in population positive for HCV in the US.

Few epidemiological studies have been done and most were limited to Asia, to determine an association between HCV and OC. The two case-control studies done in Japan have shown conflicting results [43, 44]. Nagao et al. showed increased association between HCV and OC [43]. On the other hand, Takata et al. also showed an increased risk but it turned into a protective effect after adjusting for age [44]. A large cohort study conducted by Su et al. on Chinese population showed an increased risk of OC in HCV-infected population as compared to HCV-negative population [45]. However, cohort studies done outside of Asian population has not shown an association. Until now, no definitive conclusions can be obtained from the data on the association of HCV and OC and more studies are needed utilizing information on the risk factors of OC to be adjusted in the data analyses for making more definite conclusions.

Epidemiologic studies on the relationship of HCV and BC have consistently failed to show an association [28, 34–36, 46, 47]. Only Su et al. noticed an increased risk in women aged < 50 years [46]. Thus, recent epidemiologic data on the association of HCV and BC do not confer a causative relationship. Therefore, increased screening of BC in chronic HCV infection is not warranted.

Two case-control studies done in Turkey and Italy, by Malaguarnera et al. and Uzun et al., on the association of HCV and LC have observed that HCV increases the risk of lung cancer [39, 48]. Until now, no cohort study has specifically looked into the association of HCV and LC. Although the four cohort studies have shown an increased risk of LC in HCV infected population [28, 34–36], two of them did not find the risk statistically significant [28, 35]. Currently, limited epidemiological studies have suggested some association between HCV and LC. Clinicians may consider keeping a low threshold for screening chronic HCV infection for LC until further more focused prospective studies evaluate the relationship between HCV and LC in more detail.

The data on the association of HCV and nonpancreatic GI cancers is very limited and consist mainly of a case control study done in Italy [39] and a few cohort studies based on administrative datasets [28, 34–36]. Malaguarnera et al. showed increased prevalence of anti-HCV antibodies among CRC as compared to controls [39]. Also, Allison et al. found increased incidence of rectal cancer in HCV-infected population [34]. None of the other cohort studies were able to confirm these associations. None of the cohort studies found an association between HCV and EC or SC [28, 34, 35]. Thus, it can be concluded that the current data on the association of HCV and nonpancreatic GI cancers is very limited and does not clearly indicate if there is an association.

Most of available data on the association of HCV and nonhepatic solid cancers have been obtained from cohort studies. The large size and lengthy follow-up of these studies provides the adequate statistical power to determine the cancer risk in the study population. However, lack of consistency has been found across the epidemiological studies on the association of HCV and nonhepatic solid cancers which could be explained by the limitations associated with them. These limitations include small sample size of some case-control studies, errors in diagnosis in administrative dataset, representation of only a part of the general population in the national cancer registry, and missing data. This may result in underestimation or overestimation of the risk and makes it difficult to interpret the results of these studies. For example, two case-control studies done in Turkey and Italy, by Malaguarnera et al. and Uzun et al., were limited by a small sample size [48] and not being able to adjust for risk factors of LC like smoking, radiation exposure, occupation, and family history of LC [39, 48]. Similarly, Su et al. lacked information on several variables associated with BC, for example, body mass index, fertility, and use of oral contraceptive to adjust for confounding in their analyses [46]. Woo et al. utilized paired matching to match their controls with cases for age, sex, and date of admission or visit but did not use conditional logistic regression for analyzing the data as indicated when controls are pair-matched to cases [22]. Also, there were too much missing data (66%) in this study which may have contributed to bias to their analyses [22]. Budakoğlu et al. did not confirm the anti-HCV antibody-positive status of the subjects with HCV RNA which could have resulted in a bias in their analysis since anti-HCV antibodies can be falsely positive due to old infection or secondary to cross reactivation with other infections [40]. Hofmann et al. excluded 623 HCV-infected subjects from the analyses due to incomplete information [42]. It is not known if the characteristics of the subjects excluded from the analyses were similar to those who were included and thus may have resulted in a bias [42]. A large cohort study conducted by Su et al. showed an increased risk of OC in HCV-infected population as compared to HCV-negative population [45]. However, this study also lacked information on some of the risk factors of oral cancer like smoking, alcohol consumption, and betel squid chewing to adjust them in their analyses [45]. Although Malaguarnera et al. observed an increased prevalence of HCV RNA in LC patients as compared to controls (26% versus 6.6%), they did not confirm this by using statistical analyses to look for significance [39]. Moreover, no study has looked into the role of different HCV genotypes in causing a specific type of nonhepatic solid cancer.

The data on the association between HCV and many nonhepatic solid cancers are still in the initial stages and are not very conclusive. More epidemiological studies are needed in different regions of the world to confirm the associations observed in this review.

## Figures and Tables

**Table 1:** Case-control studies for association of chronic HCV infection with pancreatic cancer (PC).

Author	Country	Sample size (*n*)	HCV (+) cases (*n*)/total cases (*n*)	HCV (+) controls (*n*)/total controls (*n*)	Factors adjusted	AOR (95% CI)	*P* value
Woo et al. [22]	Korea	3765	21/753	36/3012	Age, sex, DM, smoke	2.3 (1.3–4.0)	<0.01
Chang et al. [23]	Taiwan	2301	22/585	45/1716	Age, sex, DM, smoke	1.3 (0.8–2.3)	NA
Hasan et al. [24]	USA	1355	7/476	9/879	DM, smoke, alcohol, FHC	0.9 (0.3–2.8)	NA

AOR: adjusted odds ratio; CI: confidence interval; DM: diabetes mellitus; FHC: family history of cancer.

**Table 2:** Cohort studies for association of chronic HCV infection with pancreatic cancer (PC).

Author	Country	Study period	Total number of subjects	PC (*n*)/HCV (+) cases (*n*)	PC (*n*)/HCV (−) cases (*n*)	OC	EC	Person-years	Risk (95% CI)
Abe et al. [25]	Japan	16 years	20,360	NA/1129	NA/19,231	NA	NA	324,394	HR^∗^ 0.7 (0.3–1.7)
Allison et al. [34]	USA	2006–2010	2,143,369	19/12,126	NA/2,131,243	NA	NA	39.984	SRR 2.5 (1.7–3.2)
Huang et al. [26]	Sweden	1990–2006	50,953	34/39,442	120/197,208	34	16.5	360,154	SIR^∗∗^ 2.1 (1.4 2.9) HR 1.6 (1.0–2.4)
Omland et al. [35]	Denmark	1994–2003	4204	4/4204	NA	4	1	15,980	SIR 3.9 (1.1–10.1)
El-Serag et al. [27]	USA	1996–2004	718,687	NA/146,394	NA/572,293	NA	NA	280,676^∗∗∗^ 1,095,911^∗∗∗∗^	HR^∗∗∗∗∗^ 1.2 (1.0–1.5)
Amin et al. [28]	Australia	1990–2002	117,547	17/75,834	NA	17	NA	356,775	SIR 1.4 (0.8–2.2)

CI: confidence interval; OC: observed cases; EC: expected cases; SRR: standardized rate ratios; SIR: standardized incidence ratios; OST: opioid substitution therapy. ^∗^HR adjusted for age, sex, study area, diabetes, BMI, and smoking. ^∗∗^SIR WITH lag period after 6 months of HCV notification. ^∗∗∗^Person-years in HCV infected cohort. ^∗∗∗∗^Person-years in HCV-uninfected cohort. ^∗∗∗∗∗^HR adjusted for age, sex, baseline visit date, type of visit (inpatient or outpatient) for the baseline visit, and a preceding visit, previous VA use, and era of military service.

**Table 3:** Case-control studies for association of chronic HCV infection with thyroid cancer (TC).

Author	Country	Study design	Sample size (*n*)	HCV (+) cases (*n*)/total cases (*n*)	HCV (+) controls (*n*)/total controls (*n*)	Factors adjusted	OR (95% CI)	*P* value
Antonelli et al. [29]	Italy	Control^a^ Control^b^	1540	6/308	0/616^a^ 1/616^b^	N/A	N/A	0.001^a^ 0.003^b^
Montella et al. [30]	Italy	HCV diagnosis—ELISA and PCR	34/372	16/130	18/242	N/A	3.3 (1.5–7.4)	0.003
Antonelli et al. [31]	Italy	TC in HCV related MC	564	2/94	0/582	N/A	N/A	0.001
Montella et al. [32]	Italy	HCV prevalence in different cancers versus controls 130 TC cases	356	16/130	17/226	Age, sex	(a) Overall population 2.8 (1.2–6.3) (b) ≥50 years 3.1 (1.2–8.0)	(a) 0.01 (b) 0.01
Antonelli et al. [33]	Italy	FNA of PTN	974	3/139	0/835	N/A	N/A	0.004

CI: confidence interval; ^a^control—iodine deficient area; ^b^control—iodine sufficient area; ELISA: enzyme-linked immunosorbent assay; PCR: polymerase chain reaction; MC: mixed cryoglobulinemia patients; FNA: fine-needle aspiration; PTN: palpable thyroid nodules; NA: not available.

**Table 4:** Cohort studies for association of chronic HCV infection with thyroid cancer (TC).

Author	Country	Study period	Total number of subjects	TC (*n*)/HCV (+) cases (*n*)	TC (*n*)/HCV (−) cases (*n*)	OE	EC	Person-years	Risk (95% CI)
Duberg et al. [37]	Sweden	1990–2000	27,150	NA/27,150	NA	5	NA	122,272	SIR 1.5 (0.5–3.6)
Giordano et al. [4]	USA	1997–2004	718,687	NA/146,394	NA/572,293	NA	NA	HCV (+) 280,676 HCV (−) 1,095,911	HR^∗^ 0.7 (0.5–0.9)
Allison et al. [34]	USA	2006–2010	2,143,369	6/12,126	NA/2,131,243	NA	NA	39.984	SRR 1.1 (0.6–1.6)
Omland et al. [35]	Denmark	1994–2003	4204	1/4204	NA	1	0.5	15,980	SIR 2.1 (0.1–12.0)
Amin et al. [28]	Australia	1990–2002	117,547	9/75,834	NA	9	NA	356,775	SIR 0.3 (0.2–0.7)
Swart et al. [36]	Australia	1993–2007	29,613	5/14,892	NA	5	6.8	213,008	SIR 0.7 (0.2–1.7)

OC: observed cases; EC: expected cases; CI: confidence interval; SIR: standardized incidence ratio; HR: hazard ratio; ^∗^HR adjusted for age, sex, race, baseline visit date, type of visit (inpatient or outpatient) for the baseline visit and a preceding visit, previous VA use, and era of military service; NA: not available.

**Table 5:** Case-control studies for association of chronic HCV infection with renal cancer (RC).

Author	Country	Study design	Sample size (*n*)	HCV (+) cases/total cases (*n*)	HCV (+) controls/total controls (*n*)	OR (95% CI)	*P* value
Gonzalez et al. [38]	USA	Hospital-based RCC versus CC	240	11/140^∗^ 9/11^∗∗^	1/100^∗^ 0/1^∗∗^	24.2 (2.4–>999.9)^∗∗^	0.043^∗∗^
Malaguarnera et al. [39]	Italy	Case-control	536	8/15 (53%)	30/300 (10%)	N/A	<0.001
Budakoğlu et al. [40]	Turkey	Hospital-based cases Controls from general population	6170	15/903 (1.7%)	81/5267 (1.5%)	N/A	0.77

CC: colon cancer (controls); ^∗^cases and controls tested for anti HCV; ^∗∗^anti-HCV (+) cases and controls tested for HCV RNA; OR: odds ratio; CI: confidence interval; NA: not available.

**Table 6:** Cohort studies for association of chronic HCV infection with renal cancer (RC).

Author	Country	Study period	Total number of subjects	RC (*n*)/HCV (+) cases (n)	RC (*n*)/HCV (−) cases (*n*)	OC	EC	Person-years	Risk (95% CI)
Gordon et al. [41]	USA	1997–2006	67,063	17/3057	177/64,006	NA	NA	NA	HR 1.7 (1.0–2.9)
Hofmann et al. [42]	Sweden	1990–2006	258,000	29/43,000	NA/215,000	NA	NA	400,196	SIR 1.2 (0.8–1.7) SMR 1.2 (0.6–2.2)
Omland et al. [35]	Denmark	1994–2003	4204	4/4204	NA	4	1.1	15,980	SIR 3.6 (0.9–9.2)
Amin et al. [28]	Australia	1990–2002	117,547	18/75,834	NA	18	NA	356,775	SIR 0.5 (0.1–3.4)

OC: observed cases; EC: expected cases; CI: confidence interval; SIR: standardized incidence rate; SMR: standardized mortality rate; NA: not available.

**Table 7:** Case-control studies for association of chronic HCV infection with oral cancer (OC).

Author	Country	Study design	Sample size (*n*)	HCV (+) cases/total cases (%)	HCV (+) controls/total controls (%)	Factors adjusted	OR (95% CI)	*P* value
Nagao et al. [43]	Japan	(a) Anti-HCV ab	204	(a) 24/100 (24)	(a) 11/104 (10.6)	None	NA	(a) <0.05
		(b) HCV RNA		(b) 17/100 (17)	(b) 4/104 (3.9)			(b) <0.05
Takata et al. [44]	Japan	Anti-HCV ab^∗^ Hospital-based OC IT^∗∗^	4402	13/343	25/270	Age	0.4 (0.2–0.9)	<0.05

OR: odds ratio; CI: confidence interval; NA: not available; ^∗∗^IT—impacted teeth (controls); ^∗^anti-HCV antibodies were compared among patients with OC and IT.

**Table 8:** Cohort studies for association of chronic HCV infection with oral cancer (OC).

Author	Country	Study period	Total number of subjects	OC (*n*)/HCV (+) cases (*n*)	OC (*n*)/HCV (−) cases (*n*)	OC	EC	Person-years	Risk (95% CI)
Su et al. [45]	Taiwan	1996–2008		21/5311	147/84796	NA	NA	78,803	HR 1.9 (1.2–3.0)
Amin et al. [28]	Australia	1990–2002	117,547	19/75,834	NA	NA	NA	356,775	SIR Mouth—1.5 (0.7–3.2) Tongue—1.1 (0.5–2.4) Tonsil—2.1 (1.0–4.8)
Allison et al. [34]	Australia	1993–2007	29,613	Mouth 3/14,892 Lip 3/14,892	NA	Mouth 3 Lip 3	Mouth 0.58 Lip 2.19	213,008	SIR Mouth—5.1 (1.0–15.0) Lip—1.3 (0.28–4.00)

OC: observed cases; EC: expected cases; CI: confidence interval; SIR: standardized incidence rate; SMR: standardized mortality rate; NA: not available.
